# Lower Limb Ischemia Caused by Aortic Atherosclerosis Localized in the Horseshoe Renal Isthmus

**DOI:** 10.1002/ccr3.70232

**Published:** 2025-02-18

**Authors:** Keisuke Senda, Naoki Nagahara, Minami Inoue, Ken Nishikawa, Katsuyuki Aizawa, Takahiro Takeuchi, Yoshinori Ohtsu, Hideo Tsunemoto, Chihiro Suzuki, Satoshi Yasukochi

**Affiliations:** ^1^ Department of Cardiology Aizawa Hospital Matsumoto Japan; ^2^ Department of Cardiovascular Surgery Aizawa Hospital Matsumoto Japan

**Keywords:** aortic atherosclerosis, horseshoe kidney, horseshoe renal isthmus, intermittent claudication, Leriche syndrome, vascular disease

## Abstract

Intermittent claudication in patients with horseshoe kidneys may arise from sclerotic changes in the aorta due to compression by the renal isthmus. Vascular disease is a relatively lesser‐known extrarenal complication of horseshoe kidneys, but it warrants attention.

## 1 Case Presentation

**FIGURE 1:**
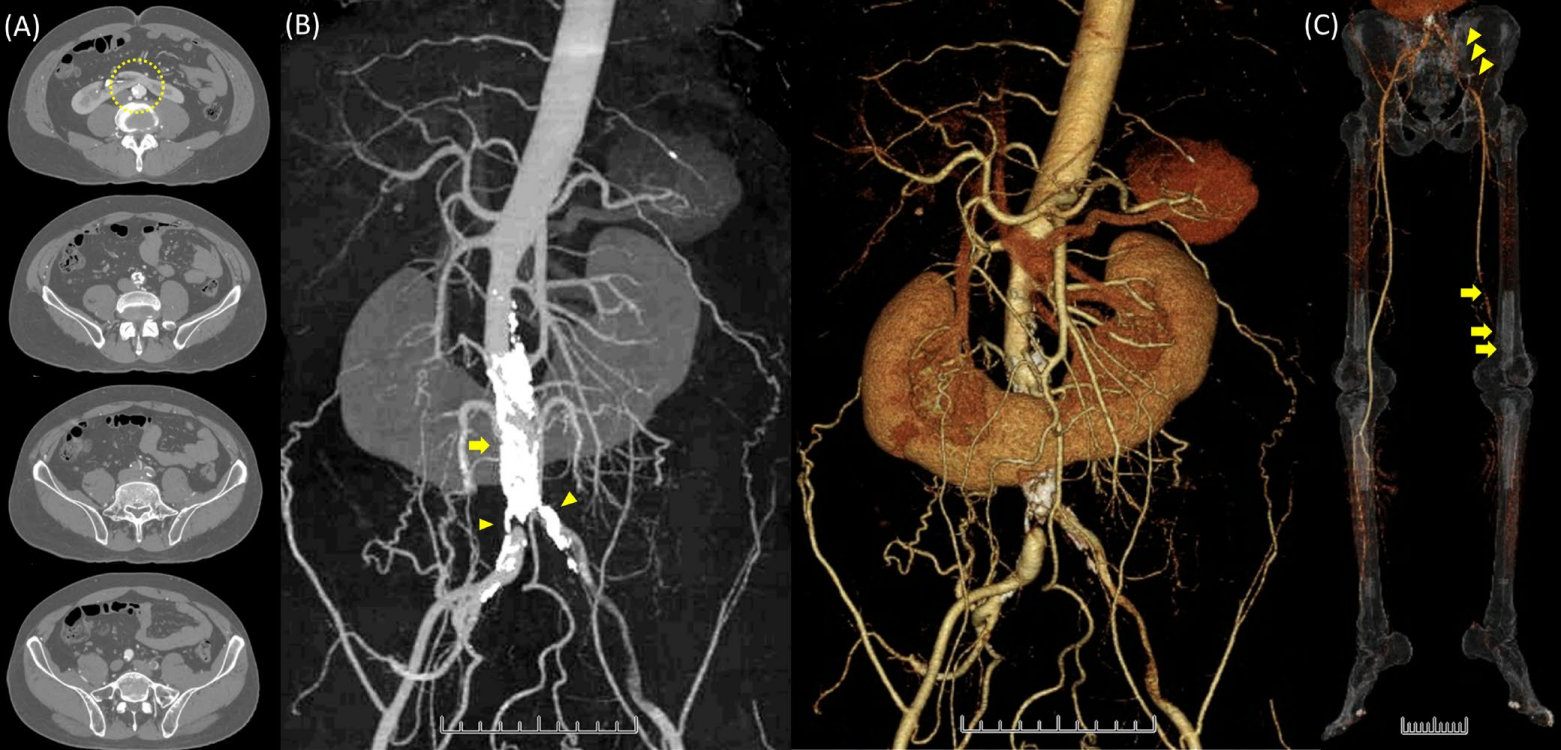
Contrast‐enhanced computed tomography demonstrated: (A) The abdominal aorta running across the horseshoe renal isthmus and the lumbar vertebrae (dotted circle). (B) Severe focal calcification of the aorta adjacent to the horseshoe renal isthmus (arrow), with occlusion of the terminal aorta and the ostia of the bilateral common iliac arteries (arrowheads). (C) Continuous thrombi observed distally (arrowheads) with prolapsed thrombi occluding the popliteal artery (arrow).

**FIGURE 2:**
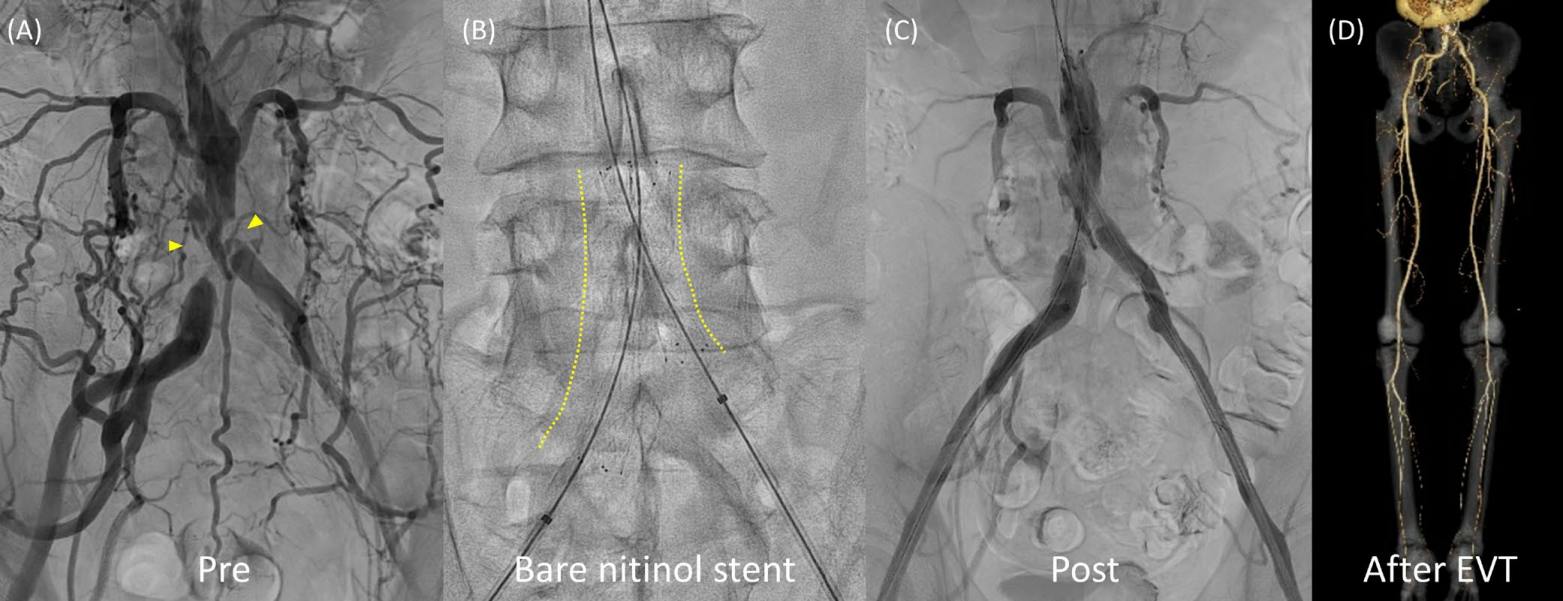
(A) Angiography showing occlusion of the terminal aorta and bilateral common iliac artery ostia (arrowheads). (B) Two bare nitinol stents deployed from the abdominal aortic terminus to the bilateral common iliac arteries in a hugging configuration. (C) Improved peripheral blood flow. (D) Urokinase administration resolving peripheral blood clots, with subsequent contrast‐enhanced computed tomography demonstrating improved blood flow to the left lower extremity.

A 57‐year‐old man with intermittent claudication presented to the hospital with a chief complaint of rest pain in the left lower extremity and difficulty walking for the past week. He was a current smoker but had no history of hypertension, diabetes, or arrhythmia. The left lower extremity appeared pale, purple, and cold, with motor and sensory disturbances in the foot. Blood tests revealed an elevated creatine kinase level. Contrast‐enhanced computed tomography showed severe focal calcification of the aorta adjacent to the horseshoe renal isthmus, and at the terminus, both common iliac arteries were occluded. Except for this part of the aorta, calcification was not significant (Figure 1A,B). On the left side, continuous thrombi were observed distally, and prolapsed thrombi occluded the popliteal artery (Figure 1C). The diagnosis was Leriche syndrome with acute exacerbation of chronic lower limb ischemia due to thrombus occlusion. Endovascular therapy (EVT) of the popliteal artery and urokinase administration were performed, followed by EVT of the bilateral common iliac arteries, resulting in symptom improvement (Figure 2).

## 2 Discussion

Leriche syndrome is characterized by terminal aortic occlusion. Its etiology has been attributed to vasculitis or atherosclerosis, with more recent reports primarily attributing it to atherosclerosis. Horseshoe kidneys are the most common fusion defect of the kidneys, occurring in approximately 0.25% of the population, with a male preponderance of 2:1 [1]. Patients with horseshoe kidneys are at an increased risk for ureteropelvic junction obstruction, nephrolithiasis, vesicoureteral reflux, urinary tract infections, transitional cell cancers, and malignant renal tumors [2]. Few associations with vascular disease have been reported, but a previous review mentioned the association between horseshoe kidney and Leriche syndrome [3]. Our case suggests that atherosclerosis of the aorta may be induced by compression from the horseshoe kidney isthmus. In this case, a focal atherosclerotic lesion with severe calcification was identified in the abdominal aorta adjacent to the isthmus of the horseshoe kidney, which progressed to Leriche syndrome, manifesting as lower limb ischemia. The horseshoe kidney may have exerted pressure on the aorta, and mechanical factors such as wall shear stress due to blood flow may have contributed to local calcification, atherosclerosis, and mural thrombus formation. Vascular disease is a lesser‐known extrarenal complication of horseshoe kidney but should not be overlooked.

## Author Contributions

**Keisuke Senda:** project administration, writing – original draft. **Naoki Nagahara:** validation. **Minami Inoue:** validation. **Ken Nishikawa:** validation. **Katsuyuki Aizawa:** validation. **Takahiro Takeuchi:** validation. **Yoshinori Ohtsu:** validation. **Hideo Tsunemoto:** validation. **Chihiro Suzuki:** supervision, writing – review and editing. **Satoshi Yasukochi:** writing – review and editing.

## Ethics Statement

The authors have nothing to report.

## Consent

Written informed consent was obtained from the patient to publish this report in accordance with the journal's patient consent policy.

## Conflicts of Interest

The authors declare no conflicts of interest.

## Data Availability

The data that support the findings of this study are available from the corresponding author upon reasonable request.
